# An Updated Review on MSMD Research Globally and A Literature Review on the Molecular Findings, Clinical Manifestations, and Treatment Approaches in China

**DOI:** 10.3389/fimmu.2022.926781

**Published:** 2022-07-18

**Authors:** Lu Xia, Xu-Hui Liu, Yuan Yuan, Douglas B. Lowrie, Xiao-Yong Fan, Tao Li, Zhi-Dong Hu, Shui-Hua Lu

**Affiliations:** ^1^ Shanghai Public Health Clinical Center, Fudan University, Shanghai, China; ^2^ Shenzhen National Clinical Research Center for Infectious Disease, Shenzhen, China; ^3^ Department of tuberculosis, The Third People’s Hospital of Shenzhen, Shenzhen, China

**Keywords:** MSMD, molecular mechanisms, immunological mechanisms, clinical characteristics, diagnosis, treatment, prognosis

## Abstract

Mendelian susceptibility to mycobacterial disease (MSMD) arises from a group of rare inherited errors of immunity that result in selective susceptibility of otherwise healthy people to clinical disease caused by low virulence strains of mycobacteria, such as *Mycobacterium bovis* Bacille Calmette-Guérin (BCG) and environmental mycobacteria. Patients have normal resistance to other pathogens and no overt abnormalities in routine immunological and hematological evaluations for primary immunodeficiencies. At least 19 genes and 34 clinical phenotypes have been identified in MSMD. However, there have been no systematic reports on the clinical characteristics and genetic backgrounds of MSMD in China. In this review, on the one hand, we summarize an update findings on molecular defects and immunological mechanisms in the field of MSMD research globally. On the other hand, we undertook a systematic review of PubMed (MEDLINE), the Cochrane Central Register of Controlled Trials (CENTRAL), Web of Science, EMBASE, CNKI, and Wanfang to identify articles published before Jan 23, 2022, to summarize the clinical characteristics, diagnosis, treatment, and prognosis of MSMD in China. All the English and Chinese publications were searched without any restriction on article types.

## 1 Introduction

Mendelian susceptibility to mycobacterial disease (MSMD) is a rare class of inherited errors of immunity that result in selective susceptibility to clinical disease caused by mycobacteria that have low virulence, such as *Mycobacterium bovis* Bacille Calmette-Guérin (BCG) vaccine and environmental non-tuberculous mycobacteria (NTM), in otherwise healthy patients. No overt immunological abnormalities are revealed in routine immunological and hematological evaluations for primary immunodeficiencies (PID). Patients with MSMD are also predisposed to invasive infections with other intracellular microorganisms, including *Mycobacterium tuberculosis* (*Mtb*) and non-mycobacterial pathogens, such as *Salmonella, Listeria, Histoplasma, Toxoplasma*, and several viruses (1, 2). Since the first genetic etiology was discovered in 1996, MSMD has been reported in more than 50 countries worldwide, with a prevalence of about 1/50,000 individuals (3). As the second-highest prevalence of tuberculosis (TB) globally, China has a population of about 1.4 billion in which all infants receive BCG vaccination. However, there have been no systematic reviews on the clinical characteristics and genetic profile of MSMD in China. In this review, we first provide a summary of updated findings on molecular defects and immunological mechanisms in the field of MSMD research globally, and then we undertook a systematic review to summarize the clinical characteristics, diagnosis, treatment, and prognosis of MSMD in China.

## 2 An Updated Findings on Molecular Defects and Immunological Mechanisms Researches in the Field of MSMD Research Globally

### 2.1 An Updated Findings on Molecular Defects Researches in MSMD

The first clinical descriptions of MSMD were two reports of idiopathic disseminated BCG infection (BCGosis) following vaccination in the late 1940s and early 1950s (4–6). In 1996, the first genetic etiology of MSMD was discovered in an infant who developed a fatal infection following BCG inoculation and it was due to bi-allelic null mutations of *IFNGR1* (7, 8). Since then, many more MSMD-causing mutations have been identified, and allelic heterogeneity at these loci has been found to result in more than 30 different genetic disorders of MSMD, distinguished by the influence of different mutations, the expression of the mutant allele, the mode of transmission, and the function affected (7, 8). Among which, mutations in *IL12RB1* are the most reported (~44%) genetic defects in MSMD, followed by *IFNGR1* (~17%) and *IL12B* (~12%). However, almost 60% of patients with clinical manifestations suggestive of MSMD lack a defined genetic cause (9). The genetic mutations known to lead to MSMD are shown in **Table 1**, ordered by the year of their first published reports, and a brief description is provided below.

**Table 1:** Overview of genetic etiologies involved in MSMD.

Gene symbol	Gene full name	Signaling affected	Inheritance	Functional impairment	OMIM ID	Chromosomal position
*IFNGR1*	Interferon-gamma receptor-1	IFN-γ	AR/AD	C/P	107470	6q23-q24
*IFNGR2*	Interferon-gamma receptor-2	IFN-γ	AR/AD	C/P	147569	21q22.11
*IL12B*	Interleukin 12B	IL-12/IL-23	AR	C	161561	5q31.1-31.1
*IL12RB1*	Interleukin 12 receptor subunit beta 1	IL-12/IL-23	AR	C	601604	19p13.1
*STAT1*	Signal transducer and activator of transcription 1	IFN-γ, IFN-α/β	AR/AD	C/P	600555	2q32.2
*NEMO/IKBKG*	NF-κB essential modulator/Inhibitor of NF-κB kinase subunit gamma	IL-12	XR	P	300248	Xq28
*TYK2*	Tyrosine kinase 2	IL-12/IL-23	AR	C	176941	19p13.2
*IRF8*	Interferon regulatory factor 8	IFN-γ	AD	P	601565	16q24.1
*CYBB*	Cytochrome b-245 beta chain	NADPH oxidase activity	XR	P	300645	Xp21.1-p11.4
*ISG15*	Interferon-stimulated gene product 15	IFN-γ	AR	C	616126	1p36.33
*RORC*	RAR related orphan receptor C	IFN-γ/IL-17	AR	C	602943	1q21.3
*JAK1*	Janus kinase 1	IFN-γ	AR	P	147795	1p31.3
*IL12RB2*	Interleukin 12 receptor subunit beta 2	IL-12	AR	C	601642	1p31.3
*IL23R*	Interleukin 23 receptor	IL-23	AR	C	607562	1p31.3
*SPPL2A*	Signal peptide peptidase-like 2A	IL-12/IL-23	AR	C	608238	15q21.2
*IFNG*	Interferon-gamma	IFN-γ	AR	C	147570	12q15
*TBX21*	T-box transcription factor 21	IFN-γ	AR	C	604895	17q21.32
*ZNFX1*	Zinc finger NFX1-type-containing 1	Stress granules recruiting	AR	C	618931	20q13.13
*PDCD1*	Programmed cell death 1	IFN-γ	AR	C	600244	2q37.3

AR, autosomal recessive; AD, autosomal dominant; XR, X-linked recessive; C, Complete; P, Partial.

#### 2.1.1 IFNGR1

The *IFNGR1* gene encoding IFN-γR1 protein provided the first-found genetic etiology of MSMD in 1996; bi-allelic null mutations resulted in IFN-γR1 deficiency (10, 11). This gene encodes the ligand-binding chain of the IFN-γ receptor. The human IFN-γ receptor is a heterodimer of IFN-γR1 (ligand-binding) and IFN-γR2 (signal-transducing) chains of the IFN-γ receptor. The human *IFNGR1* gene is located on chromosome 6q23.3 and spans an area of about 22 kb, in which more than 40 unique *IFNGR1* mutations have been reported, and they exert both autosomal recessive (AR) and autosomal dominant (AD) effects. The majority of *IFNGR1* mutations are premature stop codons in the intracellular domain of the IFN-γR1 protein that affects recycling, followed by amino acid substitutions (12, 13). The largest number of variants have been found in exon 6, all of which are AD mutations. The complete AR deficiency leads to severe, early-onset, and life-threatening infections with BCG, NTM, and other pathogens, whereas partial AD results in less severe, and later onset mycobacterial diseases (14, 15).

#### 2.1.2 IFNGR2

The *IFNGR2* gene encodes IFN-γR2 protein and is located on chromosome 21q22.11, spanning a region of 33 kb. In 1998, a child with disseminated NTM infection caused by a premature stop codon mutation in the extracellular domain of *IFNGR2* was reported. The mutation prevented protein expression on the cell surface and led to intracellular protein degradation (16). Since then, more than 20 variations have been reported in more than 20 patients (9). The majority of the variants cause complete AR deficiency of IFNGR2, and only 1 case of partial AD deficiency has been reported up to now (17). The clinical manifestations of complete deficiency of IFNGR2 are similar to those of complete IFNGR1 deficiency, and patients present with severe or even fatal infection early in life.

#### 2.1.3 *IL12B*

The *IL12B* gene encodes the 40-kD heavy chain of cytokine IL-12p70, a disulfide-linked heterodimer composed of the p40 subunit and a p35 subunit encoded by *IL12A*. IL-12 is mainly expressed by myeloid macrophage and dendritic cells (DCs) and plays a major role in inducing the production of IFN-γ by natural killer (NK) and T lymphocytes. It also participates in the IL-23 signaling pathway. Located on chromosome 5q33.3, *IL12B* comprises a sequence of about 16 kb. In 1998, a large homozygous deletion within the IL-12p40 subunit gene was first found in a child with disseminated BCG and *Salmonella enteritidis* infections (18). Such mutations always result in a complete deficiency of IL-12p40 and defects in both IL-12 and IL-23 dependent immunity. The clinical features are characterized by childhood onset of BCG and *Salmonella* infections, with relatively high levels of recurrence (19).

#### 2.1.4 *IL12RB1*

The *IL12RB1* gene encodes IL-12Rβ1, a type I transmembrane protein belonging to the hematopoetin receptor superfamily. IL-12Rβ1 forms a disulfide-linked oligomer required for IL-12 binding activity. The co-expression of IL-12Rβ1 and IL-12Rβ2 leads to the reconstitution of IL-12-dependent signaling. Mutations in *IL12RB1* impair the development of IFN-γ- and IL-17-producing T lymphocytes. IL12RB1 also binds to the receptor of IL-23, to initiate IL-23 signaling. *IL12RB1* is located on chromosome 19p13.1 and spans about 28 kb. Bi-allelic mutations of the *IL12RB1* gene result in IL-12Rβ1 deficiency. In 1998, four patients from three unrelated kindreds who suffered from disseminated mycobacterial infections, including BCG and *Mycobacterium avium complex* (MAC), were found not to be deficient in *IFNGR1* but they all had mutations in *IL12RB1* (20). Since then, almost half of the genetic defects in MSMD have been identified as AR mutations in *IL12RB1.* More than 100 unique variants have been reported, with a heterogeneous clinical presentation ranging from infantile death to being asymptomatic until adulthood (21).

#### 2.1.5 *STAT1*

The *STAT1* gene encodes a member of the cell signal transducer and activator of transcription (STAT) protein family and spans a length of 57 kb on chromosome 2q32.2. In response to cytokines and growth factors, STAT family members are phosphorylated by the receptor-associated kinases and then form homodimers or heterodimers that translocate to the cell nucleus, where they act as transcription activators. STAT1 is a transcription factor involved in the cell viability support mediated by cytokines, including interferons of types I (IFN-α/β), II (IFN-γ), III (IFN-λ), and IL-27. STAT1 participates in IFN-γ signaling in anti-mycobacterial immunity and in the IFN-α/β signaling of anti-viral immunity. Different patterns of inheritance of STAT1 deficiency have been described in humans: bi-allelic mutations cause complete (22–24) or partial AR deficiency (25), whereas mono-allelic mutations cause AD deficiency (26). In 2001, the first STAT1 deficiency with AD was reported in two kindreds with susceptibility to mycobacterial but not viral diseases (26). AR complete/partial STAT1 deficiencies were first described in 2003 (24) and 2009 (25), and the patients displayed a clinical phenotype of severe mycobacterial and life-threatening viral disease, respectively. The selective susceptibility to mycobacterial infection in AD deficiency is consistent with the vital role of IFN-γ signaling in mycobacterial immunity. In contrast, the susceptibility to viral infections in AR deficiency could be explained by an impaired STAT1-dependent response to IFN-α/β.

#### 2.1.6 NEMO/IKBKG

The *NEMO*/*IKBKG* gene encodes the NF-κB essential modulator (also termed inhibitor of NF-κB kinase subunit gamma), which activates the ubiquitous transcription factor NF-κB resulting in the activation of genes involved in inflammation, cell survival, and other pathways. Located on chromosome Xq28, *NEMO* spans about 27 kb. Mutations in this gene result in incontinentia pigmenti, hypohidrotic ectodermal dysplasia, and several other types of immunodeficiencies. In 2006, the first *NEMO* deficiency was X-linked recessive (XR) and was reported in six patients from three countries. An intrinsic defect in T cell-dependent IL-12 production was observed, mediated by the impairment of the CD40-CD40L signaling pathways and resulting in defective IFN-γ secretion by T cells (27). The majority of patients with XR-*NEMO* deficiency suffered from disseminated mycobacterial diseases. However, recurrent infection with *Haemophilus influenzae* and several other Gram-negative bacteria was also reported (28).

#### 2.1.7 *TYK2*

The *TYK2* gene encodes a member of the tyrosine kinase, and more specifically the Janus kinase (JAK), protein families. This protein is associated with the cytoplasmic domain of type I and type II cytokine receptors and promulgates cytokine signals by phosphorylating receptor subunits. It is also a component of both the types I and III IFN signaling pathways. *TYK2* is located on chromosome 19p13.2 and spans about 30 kb. In 2006, the *TYK2* defect was firstly identified in a Japanese male with the hyper-IgE syndrome. He was diagnosed at the age of 22 months with a BCG infection. In addition to elevations of serum IgE, atopic dermatitis and a variety of infections including non-typhoidal *Salmonella* gastroenteritis were also found (29). Defects in the IL-12 and IL-23 signaling pathways were found in the patients with AR complete *TYK2* deficiency (30). The clinical manifestations included candidal, staphylococcal, viral, and mycobacterial infections in addition to elevated IgE.

#### 2.1.8 *IRF8*

The *IRF8* gene encodes IFN regulatory factor (IRF)-8. Proteins of this family are composed of a conserved DNA-binding domain in the N-terminal region and a divergent C-terminal region that serves as the regulatory domain., IRF proteins regulate the expression of genes stimulated by type I IFNs by binding to the IFN-stimulated response element. *IRF8* is located on chromosome 16q24.1 and spans 23 kb. In 2011, an infant presenting early-onset disseminated BCG disease and two otherwise healthy adults with a history of disseminated but curable BCG disease in childhood were reported to bear an IRF defect. The defect impaired IRF8 transcriptional activity by disrupting the interaction between IRF8 and DNA (31). The bi-allelic K108E mutation of *IRF8* caused an AR severe immunodeficiency in which there was a complete absence of circulating monocytes and DCs from peripheral blood, which resulted in disseminated BCGosis, oral candidiasis, and viral infections. A mono-allelic T80A mutation of *IRF8* was associated with a milder AD immunodeficiency and a partial reduction of CD11c^+^/CD1c^+^ circulating DCs, which led to BCGosis without other infectious diseases (32).

#### 2.1.9 *CYBB*

The *CYBB* gene encodes the β-chain of flavocytochrome b_558_. Expressed in granulocytes, monocytes, macrophages, and DCs, the protein is a crucial component of the NADPH oxidase complex. *CYBB* spans about 34 kb on chromosome Xp21.1 and deficiency can cause chronic granulomatosis disease (CGD). In 2011, an immunodeficiency without CGD features that was caused by a partial XR *CYBB* gene defect was described in seven male patients who developed BCGosis, recurrent BCG, or *Mtb* infections in two unrelated kindreds. An impaired respiratory burst was demonstrated in macrophages and B cells, but not in monocytes or granulocytes, and resulted from a cell-specific restriction of the assembly of the NADPH oxidase (33).

#### 2.1.10 *ISG15*

The *ISG15* gene encodes IFN-stimulated gene product 15, a ubiquitin-like protein that conjugates to intracellular target proteins upon activation by IFN-α and IFN-β. Expressed on neutrophils and myeloid cells, ISG15 protein can be released by bacterial challenge, inducing IFN-γ production in synergy with IL-12. *ISG15* is located on chromosome 1p36.33 and spans about 13 kb. In 2012, an AR complete *ISG15* deficiency was found in three patients with MSMD who suffered from BCG disease, but not viral infections (34). The lack of intracellular ISG15 production reduced the production of IFN-γ by lymphocytes, accounting for the enhanced susceptibility to mycobacterial disease, and could be compensated by the addition of extracellular recombinant human ISG15 protein. Another intriguing clinical phenotype of *ISG15* deficiency is intracranial calcification which may be clinically asymptomatic (35).

#### 2.1.11 *RORC*

The *RORC* gene encodes two isoforms: the ubiquitously expressed RORγ, and RORγT, which is restricted to expression on leukocytes. Located on chromosome 1q21 and spanning 26 kb, *RORC* is essential for lymphoid organogenesis and plays an important regulatory role in thymopoiesis. In 2015, AR bi-allelic RORC mutations were identified in seven individuals who had both candida and mycobacterial infections in infancy and who were from three kindreds of different ethnic origins (36). The lack of functional RORγ and RORγT isoforms led to the absence of IL-17-producing T cells and an impaired IFN-γ in response to BCG in these individuals.

#### 2.1.12 *JAK1*

The *JAK1* gene encodes a membrane protein that is a member of a class of protein-tyrosine kinases that add a phosphate group to tyrosine amino acid on substrate proteins. JAK1 protein phosphorylates STAT proteins and plays a crucial role in cytokine signal transduction. *JAK1* is located on chromosome 1p31.3. In 2016, two homozygous variants of the pseudokinase domain in *JAK1* were identified in a patient of Pakistani descent who had a mycobacterial infection and a history of recurrent viral, fungal, and parasitic skin infections since infancy along with global developmental delay. The partial AR *JAK1* deficiency caused MSMD due to defective IFN-γ responses and caused increased susceptibility to other infections as a result of impairment of type I IFN signaling. This condition may also cause susceptibility to early-onset cancer (37).

#### 2.1.13 *IL12RB2*

The *IL12RB2* gene encodes a type I transmembrane protein, IL-12Rβ2, identified as another subunit of the IL-12 receptor complex besides IL12RB1. As mentioned above, co-expression of these subunits leads to the induction of IL-12-dependent signaling. The gene is located on chromosome 1p31.3. In 2018, a homozygous mutation was identified in *IL12RB2* in a consanguineous Turkish family (38). Production of the truncated protein caused complete impairment of the IL-12 signaling pathway due to the lack of the transmembrane segment, and inheritance was AR. This study additionally indicated that the relative rarity of symptomatic patients with *IL-12RB2* deficiency relative to *IL-12RB1* deficiency was not because of differences in the frequency of the genetic disorders, but due to lower clinical penetrance: the isolated absence of *IL-12RB2* was, at least in part, compensated by other cytokines for the production of IFN-γ, which thereby provided some protection against mycobacteria. In contrast, the deficiency of *IL-12RB1* abolished both IL-12 and IL-23 immunity. Thus, both IL-12 and IL-23 are required for optimal IFN-γ-dependent immune responses to mycobacterial infection, effective individually but much more so cooperatively.

#### 2.1.14 *IL23R*

The *IL23R* gene encodes a subunit of the receptor for IL-23 protein that pairs with IL12RB1 protein in initiating IL-23 signaling. IL-23 protein associates constitutively with JAK2, and binds to transcription activator STAT3 in a ligand-dependent manner. *IL23R* is located on chromosome 1p31.3 within 150 kb of the gene for *IL-12RB2*. In 2018, a homozygous mutation of *IL23R*, which abolished IL-23 cellular response, was identified in two siblings in consanguineous Iranian kindred (38). Another study reported three cases of *IL-23R* deficiency in Iranian patients with MSMD (39). Similar to deficiency of *IL12RB2*, *IL-23R* deficiency displayed a low clinical penetrance due to the IL-12-mediated compensatory pathway in IFN-γ-dependent immunity to mycobacteria.

#### 2.1.15 *SPPL2A*

The *SPPL2A* gene encodes signal peptide peptidase-like 2A, which is a transmembrane protease that is expressed in all major adult human tissues and localizes to late endosomal compartments and lysosomal membranes. This membrane-bound protease degrades the N-terminal fragment (NTF) of the human leukocyte antigen (HLA) invariant chain CD74. *SPPL2A* is located on chromosome 15q21.2 and spans about 63 kb. In 2018, *SPPL2A* mutation was identified in three patients from two kindreds presenting BCG disease a few months after vaccination (40). In these patients with AR complete deficiency, CD74 NTF accumulated in the HLA class II myeloid and lymphoid cells, which selectively depleted IL-12- and IL-23-producing CD1c^+^ DCs and their circulating progenitors. Moreover, *SPPL2A*-deficient memory T cells failed to secrete IFN-γ after mycobacterial antigen stimulation.

#### 2.1.16 *IFNG*

The *IFNG* gene encodes IFN-γ, a homodimer that binds to its receptor to trigger a cellular response to microbial infections. Located on chromosome 12q15, *IFNG* contains about 5 kb. In 2020, two cousins with a homozygous deletion in *IFNG*, which caused a premature stop codon, were reported (41). These two cousins had disseminated BCGosis due to complete IFN-γ deficiency, and no other symptomatic bacterial and viral infections were detected.

#### 2.1.17 *TBX21*

The *TBX21* gene is a member of a phylogenetically conserved family of genes that share a common DNA-binding domain, the T-box, which is the human ortholog of the murine *Tbet* gene. Studies in mice showed that T-bet protein is a Th1 cell-specific transcription factor that controls the expression of the hallmark Th1 cytokines, especially IFN-γ, suggesting a role for this gene in initiating Th1 lineage development from naive Th precursor cells. Located on chromosome 17q21.32, *TBX21* contains about 58 kb. In 2020, an AR status with complete *TBX21* deficiency was identified in a three-year-old boy who suffered from BCGosis following vaccination at three months and had persistent reactive airway disease but was otherwise healthy (42). Impaired production of IFN-γ by innate and innate-like lymphocytes was observed in the patient.

#### 2.1.18 *ZNFX1*

The *ZNFX1* gene encodes NFX1-type zinc-finger–containing-1, a highly conserved IFN-stimulated sensor of double-stranded RNA that restricts the replication of RNA viruses in mice. It is ubiquitously expressed at a low level in uninfected cells but is rapidly upregulated in response to viral infections and exposure to type I IFNs. The *ZNFX1* protein binds to viral RNA and interacts with the mitochondrial antiviral signaling protein, promoting the expression of IFN-stimulated genes in mouse models. However, its function in the human immune response remains elusive. *ZNFX1* is located on chromosome 20q13.13. In 2021, an AR *ZNFX1* deficiency was identified in thirteen patients from eight unrelated kindreds with severe infections by both RNA and DNA viruses and virally triggered inflammatory episodes (43). In the same year, an AR *ZNFX1* deficiency was identified in four patients from two unrelated kindreds with intermittent monocytosis and mycobacterial disease, including BCGosis and disseminated TB, and without any known inborn IFN-γ error or severe viral illnesses (44). The study indicated that human *ZNFX1* might be essential for monocyte homeostasis and protective immunity against mycobacteria through stress granule recruiting mechanisms potentially involving myeloid cells. However, more studies are warranted to delineate more clearly the functions of human *ZNFX1* in anti-mycobacterial immunity. In addition, other clinical manifestations probably remain to be documented as more *ZNFX1*-deficient patients are characterized in the future.

#### 2.1.19 *PDCD1*

The *PDCD1* gene codes programmed cell death protein (PD-1), which is an immune-inhibitory receptor expressed in activated T cells and is involved in the regulation of T-cell functions. The protein can also promote the differentiation of CD4^+^ T cells into T regulatory cells. *PDCD1* is located on chromosome 2q37.3. In 2021, a homozygous frameshift variant of *PDCD1* was identified in a child who was born to consanguineous Turkish parents and suffered from severe TB and autoimmunity. The patient’s leukocytes did not express PD-1 and secreted only small amounts of IFN-γ upon mycobacterial stimulation, a similarity to inborn errors of IFN-γ production (45). More studies are warranted to clarify the role of *PDCD1* in immunity against other mycobacterial infections besides TB.

### 2.2 Immunological Mechanisms of MSMD

As described above, most of the mutated genes in patients with MSMD are involved in the IFN-γ/IL-12/IL-23 signaling, collectively demonstrating the importance of this signaling axis in anti-mycobacterial immunity. When infection is initiated, the pathogen-associated molecular patterns on mycobacteria are recognized *via* various phagocyte-expressed pattern-recognition receptors to facilitate phagocytic uptake of the bacteria and result in the production of IL-12, IL-23, and other cytokines (46, 47). By binding with its receptor, IL-23 induces early production of IFN-γ in synergy with IL-1β or IL-18. IFN-γ binds to its receptor in macrophages, resulting in the activation of JAK1/JAK2 signaling and STAT1 phosphorylation. The homodimers of phosphorylated STAT1 are translocated into the nucleus to induce transcription of host defense genes and the secretion of inflammatory cytokines, including IL-12 and IL-18 (48, 49). Significantly, IL-12 can compensate for an impairment of IL-23, and *vice versa*, in most humans. Consequently, the MSMD penetrance is about 50% when both cytokines are defective, whereas it is probably no higher than 0.5% when only one of these cytokines is defective (2). These are innate immune responses and help early control or even clearance of mycobacteria-infected macrophages in the early phase of infection.

Once the infecting mycobacteria break through the innate immunity barrier, T-helper 1 (Th1) cellular responses dominate the immune protection against these intracellular microbes. The antigen-presenting cells migrate to the draining lymph nodes and present foreign antigens in the context of the major histocompatibility complex to the T cell receptors expressed on T cells. This initiates the antigen-specific adaptive immune responses (50, 51). During this process, IL-12 binds to its receptor on effector T cells to activate the JAK2/TYK2 signaling, consequent to STAT4 phosphorylation, dimerization, and translocation to the nucleus to initiate the activation, proliferation, and differentiation of CD4^+^ T cells into the Th1 pathway. Similarly, IL-23 binds to its receptor on effector T cells to initiate transcription of IL-17 and IL-21 in association with IRF4 and RORγt (52, 53). The Th1 and Th17 T cell immune responses are essential for controlling the chronic phase of mycobacterial infection. In addition, in synergy with IL-18, IL-12 also induces the production of IFN-γ in natural killer and natural killer-like T cells (54). Individuals deficient in these pathways also show impaired anti-mycobacterial adaptive immunity.

**Figure 1:**
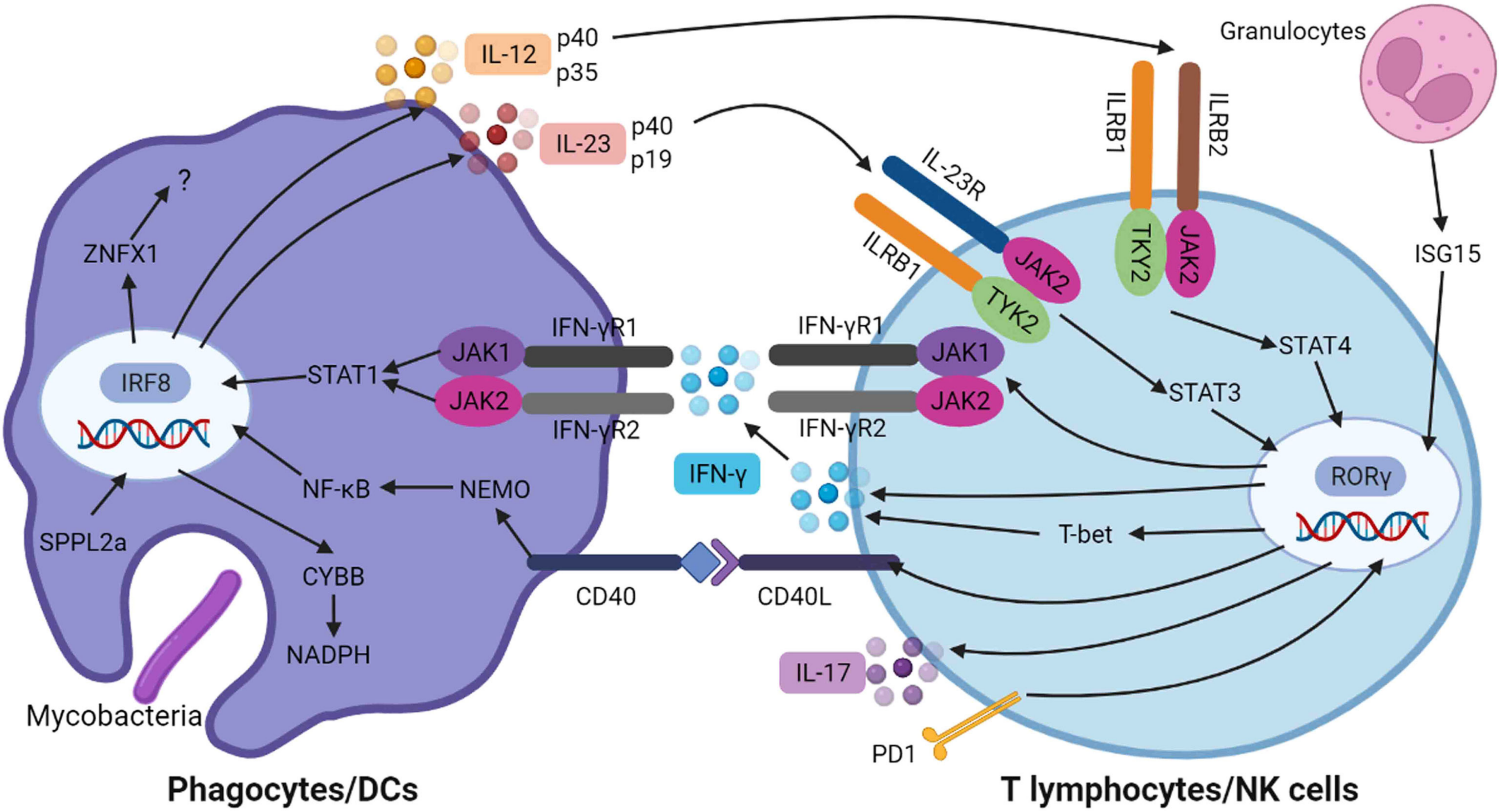
Genetic spectrum and immunological mechanisms of MSMD. When mycobacteria infection is initiated, the pathogen-associated molecular patterns are recognized *via* various phagocyte-expressed pattern-recognition receptors to facilitate phagocytic uptake of the bacteria and result in the production of IL-12 and IL-23, which bind to their receptors (ILRB1/ILRB2/IL-23R) on effector T cells leading to activation of TYK2/JAK2 signaling, consequent to STAT4 and STAT3 phosphorylation and dimerization, respectively. CYBB is required to the assembly of NADPH oxidase complex. These signaling pathways induced the transcription of IFN-γ and IL-17, which are mediated by RORγ. The IFN-γ is secreted and binds to its receptor on phagocytes, resulting in the activation of JAK1/JAK2 and STAT1 phosphorylation and dimerization, which then translocate to nucleus, resulting in the transcription of host defense genes. Meanwhile, the interaction of CD40 and its ligand result in the activation of NEMO and NF-κB mediated inflammation. This figure was created with BioRender.com.

Taken together, the monogenic defects in crucial genes involved in the IFN-γ/IL-12/IL-23 signaling axis can lead to impaired innate and/or adaptive immune responses against intracellular pathogens, resulting in the clinical phenotypes of MSMD (**Figure 1**).

## 3 Genetic Profiles and Clinical Manifestations of Reported MSMD Cases in China and Other Regions

We searched all English and Chinese articles published before Jan 23, 2022, in PubMed (MEDLINE), the Cochrane Central Register of Controlled Trials (CENTRAL), Web of Science, EMBASE, CNKI, and Wanfang databases. Keyword lists contained “MSMD” or “Mendelian susceptibility to mycobacterial diseases”, “PID” or “primary immunodeficiency disease,” and “BCG adverse reactions” in all fields.

**Figure 2:**
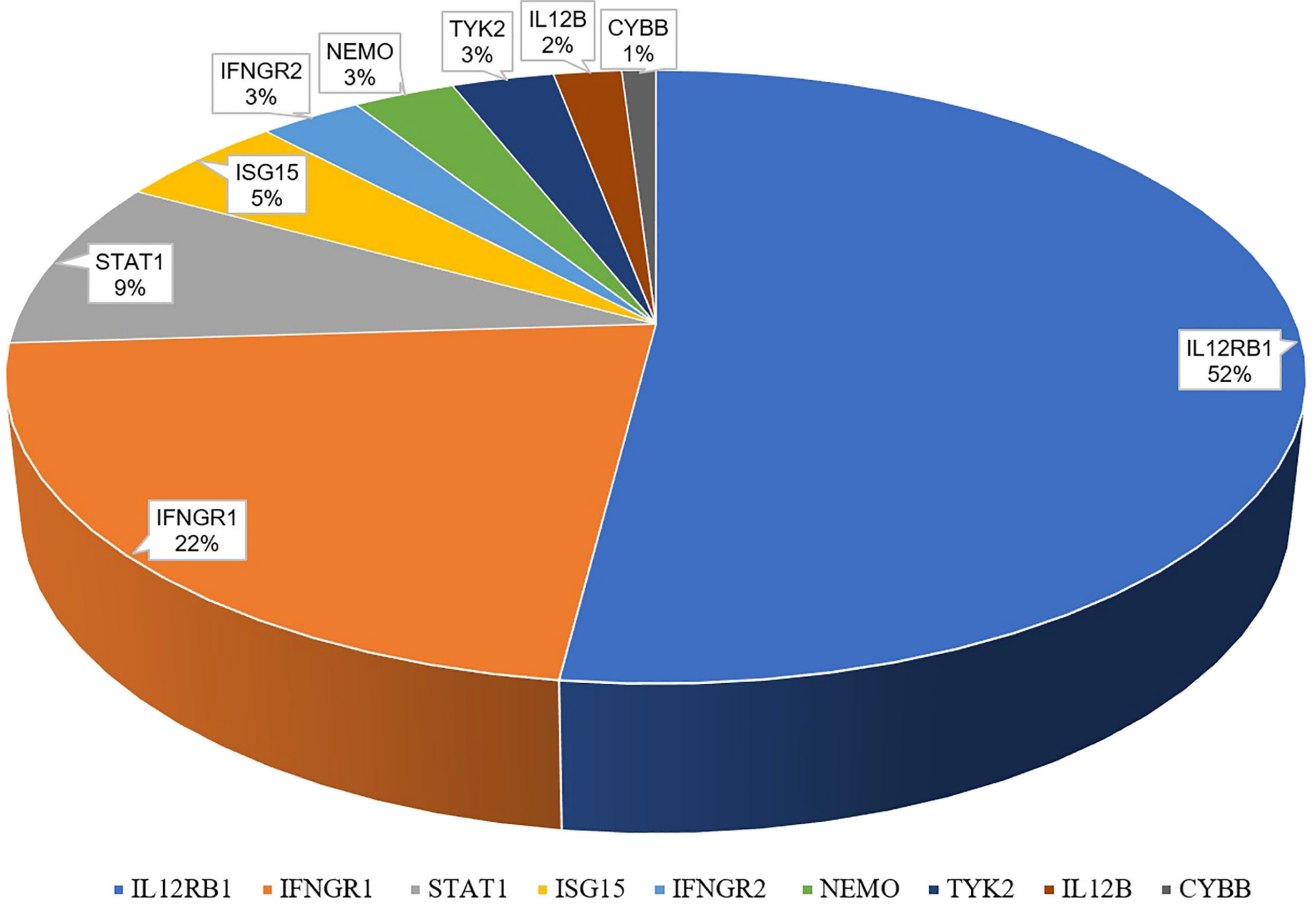
Genetic mutations of 65 Chinese MSMD patients.

**Figure 3:**
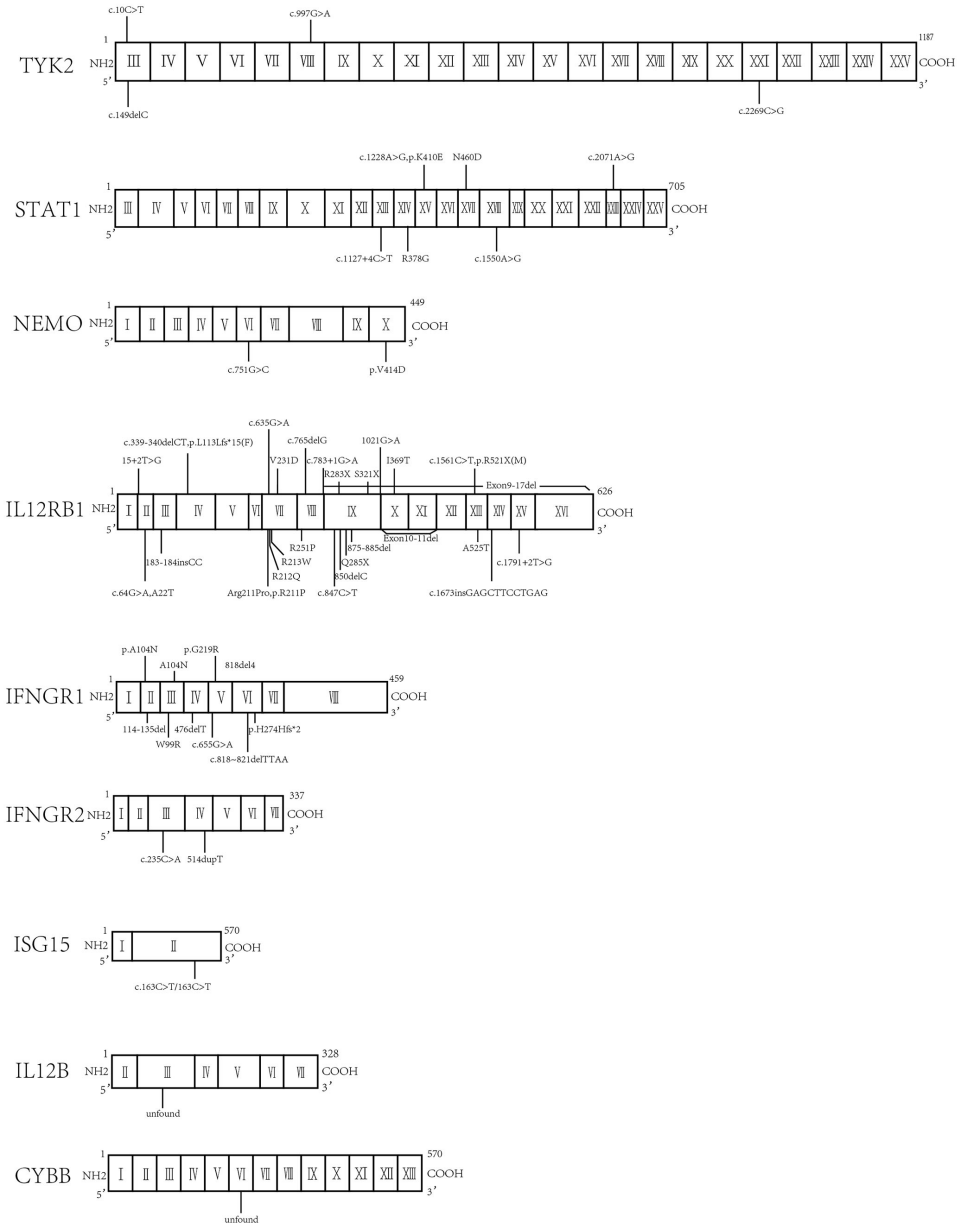
Molecular finding of all the mutations in Chinese patients.

Globally, a wide range of clinical manifestations of MSMD, from transient localized to persistent disseminated infections with impaired granuloma formation, has been reported, and *Salmonella* infections occurred in about half the reported patients (43). In China, 65 patients with MSMD in 20 publications (35, 55–72) were described, along with 9 gene mutations (*IFNGR1*, *IFNGR2*, *ISG15*, *IL12B*, *IL12RB1*, *STAT1*, *TYK2*, *NEMO*, *CYBB*) were noted. IL-12RB1 deficiency was the most common molecular defect, accounting for 52% of all cases, followed by IFNGR1 (22%) and STAT1 (9%) (**Figure 2**). The clinical manifestations and genetic mutations of all patients are presented in **Table 2** and **Figure 3**. Definitions of regional BCG-lymphadenitis (BCGitis) and BCGosis were consistent with a previous study (74).

**Table 2:** Demographic data of the 65 MSMD patients reported in China.

Patient No.	Sex	Age of onset	BCG vaccine	BCG diseases	Gene	Inheritance	Mutation site	Novel	IFN-γ therapy	Prognosis	Authors
P1	male	18y	Yes	No	*TYK2*	AR	exon7 (c.997G>A), exon3 (c.10C>T)	No	Yes	Survive	Yang Y et al (55)
P2	male	15m	Yes	No	*TYK2*	AR	exon 16(c. 2269 C>G), exon 3(c. 149 delC)	No	No	Survive	He TY et al (56)
P3	male	N/A	Yes	BCGosis	*STAT1*	AD	exon 15(c.1228A>G, p.K410E)	Yes	N/A	N/A	Liu WQ (57)
P4	female	3m	Yes	BCGosis	*STAT1*	AD	exon23 (c.2071A>G)	No	N/A	N/A	Tan Y (58)
P5	female	4m	Yes	BCGosis	*STAT1*	AD	DNA-binding (R378T)	No	No	Survive	Chen XM (59)
P6	male	5.5m	Yes	BCGosis	*STAT1*	AD	exon13(c.1127+4 C > T), exon18(c.1550A > G)	No	No	Survive	Fei L et al (60)
P7	male	1m	Yes	BCGosis	*NEMO*	XR	exon10 (p.V414D)	Yes	N/A	Died	Li L (61)
P8	male	3.5m	Yes	BCGitis	*NEMO*	XR	c.751G>C	No	N/A	N/A	Tan Y (58)
P9	male	4m	Yes	No	*IL12RB1*	AD	Arg211Pro	No	Yes	Survive	Wen-I Lee et al (62)
P10	female	1.5m	Yes	BCGosis	*IL12RB1*	AR	exon 9(Q285X)	Yes	No	Died	P. P. W. Lee et al (63) Xie N (64)
P11	female	11m	Yes	BCGosis	*IL12RB1*	N/A	N/A	N/A	No	Died	Ma WX (65)
P12	male	11m	Yes	BCGosis	*IL12RB1*	N/A	c.1561C>T, p.R521X, c.339-340 del CT, p.L113 Lfs* 15	Yes	Yes	Died	Wei QJ et al (66)
P13	male	2m	Yes	BCGosis	*IL12RB1*	AR	c.1791+2T>G	No	N/A	N/A	Gao H (67)
P14	female	2m	Yes	BCGitis	*IL12RB1*	AR	c.1791+2T>G	No	No	Survive	P. P. W. Lee et al (63)
P15	male	13m	Yes	BCGosis	*IL12RB1*	N/A	c.1791+2T>G, c.632 G>C, p.R211 P	No	No	Survive	Wei QJ et al (66)
P16	female	4m	Yes	BCGitis	*IL12RB1*	AR	c.635G > A, c.765delG	Yes	No	Survive	Zhou XP et al (68)
P17	female	3m	Yes	BCGitis	*IL12RB1*	AR	c.632G > C, c.847C > T	Yes	No	Survive	Zhou XP et al (68)
P18	male	8m	Yes	BCGitis	*IL12RB1*	AR	exon 14(c.64G > A, c.1673insGAGCTTCCTGAG)	Yes	No	Survive	Zhou XP et al (68)
P19	female	20m	Yes	BCGitis	*IL12RB1*	AD	exon7(R211P), exon10(I369T)	No	Yes	Survive	Yu XL et al (69)
P20	male	5y	Yes	BCGosis	*IL12RB1*	AR	exon 8(c.783+1G>A)	No	Yes	Survive	Li L (61)
P21	male	1m	Yes	BCGitis	*IL12B*	AR	exon3(unfound)	N/A	No	Died	Li L (61)
P22	male	2.5m	Yes	BCGitis	*IFNGR2*	AD	c. 235C>A	No	N/A	N/A	Tan Y (58)
P23	female	11m	Yes	BCGosis	*IFNGR1*	AD	exon 6(818del4)	No	No	Died	Xu H et al (70)
P24	female	1m	Yes	BCGitis	*IFNGR1*	N/A	c.818-821delTTAA	No	No	N/A	Tan Y (58)
P25	female	3m	Yes	BCGosis	*IFNGR1*	AD	c.655G>A	No	N/A	N/A	Gao H (67)
P26	female	<1y	Yes	BCGitis	*IFNGR1*	AD	exon 6(818del4)	No	No	Survive	Wen-I Lee et al (62)
P27	female	8m	Yes	BCGitis	*IFNGR1*	AD	exon 6(p.N274Hfs*2)	No	No	Survive	Xiao M et al (71)
P28	female	3m	Yes	BCGosis	*IFNGR1*	AR	p.G219R	No	Yes	Survive	Ying WJ et al (72)
P29	female	2m	Yes	BCGosis	*IFNGR1*	AR	p.G219R, p.A104N	Yes	Yes	Survive	Ying WJ et al (72)
P30	male	1m	Yes	BCGosis	*IFNGR1*	AD	exon 6(818del4)	No	Yes	Survive	Wen-I Lee et al (62)
P31	female	8m	Yes	BCGosis	*IFNGR1*	AD	exon 6(818del4)	No	Yes	Survive	Wen-I Lee et al (62)
P32	male	8m	Yes	BCGosis	*CYBB*	XR	exon5(unfound)	N/A	No	Survive	Li L (61)
P33	female	13y	No	No	*ISG15*	AR	exon2(c.163C>T,163C>T)	No	N/A	Died	Zhang XQ et al (35)
P34	female	11y	No	No	*ISG15*	AR	exon2(c.163C>T/163C>T)	No	N/A	Survive	Zhang XQ et al (35)
P35	female	13y	No	No	*ISG15*	AR	exon2(c.163C>T/163C>T)	No	N/A	Survive	Zhang XQ et al (35)
P36	N/A	2.5m	Yes	BCGosis	*IL12RB1*	N/A	N/A	N/A	No	Died	Ying WJ et al (73)
P37	N/A	2m	Yes	BCGitis	*IL12RB1*	N/A	N/A	N/A	Yes	Survive	Ying WJ et al (73)
P38	N/A	3m	Yes	BCGosis	*IL12RB1*	N/A	N/A	N/A	No	Survive	Ying WJ et al (73)
P39	N/A	3m	Yes	BCGitis	*IL12RB1*	N/A	N/A	N/A	N/A	Lost	Ying WJ et al (73)
P40	N/A	3m	Yes	BCGitis	*IL12RB1*	N/A	N/A	N/A	N/A	Lost	Ying WJ et al (73)
P41	N/A	4m	Yes	BCGosis	*IL12RB1*	N/A	N/A	N/A	Yes	Survive	Ying WJ et al (73)
P42	N/A	3m	Yes	BCGosis	*IL12RB1*	N/A	N/A	N/A	N/A	Lost	Ying WJ et al (73)
P43	N/A	2.5m	Yes	BCGosis	*IL12RB1*	N/A	N/A	N/A	Yes	Survive	Ying WJ et al (73)
P44	N/A	2m	Yes	BCGosis	*IL12RB1*	N/A	N/A	N/A	No	Died	Ying WJ et al (73)
P45	N/A	2m	Yes	BCGosis	*IL12RB1*	N/A	N/A	N/A	Yes	Survive	Ying WJ et al (73)
P46	N/A	4m	Yes	BCGosis	*IL12RB1*	N/A	N/A	N/A	Yes	Survive	Ying WJ et al (73)
P47	N/A	3m	Yes	BCGitis	*IL12RB1*	N/A	N/A	N/A	Yes	Survive	Ying WJ et al (73)
P48	N/A	2m	Yes	BCGosis	*IL12RB1*	N/A	N/A	N/A	Yes	Survive	Ying WJ et al (73)
P49	N/A	3m	Yes	BCGosis	*IL12RB1*	N/A	N/A	N/A	Yes	Survive	Ying WJ et al (73)
P50	N/A	3m	Yes	BCGitis	*IL12RB1*	N/A	N/A	N/A	Yes	Survive	Ying WJ et al (73)
P51	N/A	4m	Yes	BCGosis	*IL12RB1*	N/A	N/A	N/A	Yes	Survive	Ying WJ et al (73)
P52	N/A	3m	Yes	BCGosis	*IL12RB1*	N/A	N/A	N/A	Yes	Died	Ying WJ et al (73)
P53	N/A	4m	Yes	BCGosis	*IL12RB1*	N/A	N/A	N/A	Yes	Survive	Ying WJ et al (73)
P54	N/A	3m	Yes	BCGitis	*IL12RB1*	N/A	N/A	N/A	Yes	Survive	Ying WJ et al (73)
P55	N/A	2m	Yes	BCGitis	*IL12RB1*	N/A	N/A	N/A	Yes	Survive	Ying WJ et al (73)
P56	N/A	4m	Yes	BCGosis	*IL12RB1*	N/A	N/A	N/A	Yes	Survive	Ying WJ et al (73)
P57	N/A	3m	Yes	BCGosis	*IL12RB1*	N/A	N/A	N/A	Yes	Survive	Ying WJ et al (73)
P58	N/A	2m	Yes	BCGosis	*IFNGR1*	N/A	N/A	N/A	No	HSCT	Ying WJ et al (73)
P59	N/A	3m	Yes	BCGosis	*IFNGR1*	N/A	N/A	N/A	Yes	Survive	Ying WJ et al (73)
P60	N/A	19m	Yes	BCGosis	*IFNGR1*	N/A	N/A	N/A	Yes	HSCT Died	Ying WJ et al (73)
P61	N/A	1.5m	Yes	BCGosis	*IFNGR1*	N/A	N/A	N/A	Yes	Died	Ying WJ et al (73)
P62	N/A	3m	Yes	BCGitis	*IFNGR1*	N/A	N/A	N/A	No	Survive	Ying WJ et al (73)
P63	N/A	2.5m	Yes	BCGosis	*IFNGR2*	N/A	N/A	N/A	Yes	Survive	Ying WJ et al (73)
P64	N/A	2m	Yes	BCGosis	*STAT1*	N/A	N/A	N/A	Yes	Survive	Ying WJ et al (73)
P65	N/A	1m	Yes	BCGosis	*STAT1*	N/A	N/A	N/A	No	Survive	Ying WJ et al (73)

BCGitis: regional BCG infection site infection; BCGosis: systemic symptoms such as fever or subfebrile status, weight loss, or stunted growth, and ≥2 areas of involvement beyond the site of BCG vaccination; AR, autosomal recessive; AD, autosomal dominant; XR, X-linked recessive; N/A, not available.

The largest cohort studied was one of 30 children in mainland China diagnosed with MSMD and followed up for 8 years (73). Among them, 22 (73.3%) had *IL12RB1* mutations, five (16.7%) had *IFNGR1* mutations, two (6.7%) had *STAT1* mutations, and one (3.3%) had *IFNGR2* mutation. Eight patients (26.7%) presented with BCGitis and the remainder (22, 73.3%) presented with BCGosis; the medium age at onset was 3 months. Co-infection was common in these cases: four (13.4%) patients developed recurrent thrush caused by *Candida albicans*; two (6.7%) pneumonia caused by klebsiella; two (6.7%) *S. enteritidis* infection; two (6.7%) skin abscesses with positive culture for *Escherichia coli* and *Staphylococcus aureus*; one (3.3%) peritonitis and one (3.3%) osteomyelitis caused by NTM.

The other 35 Chinese cases were reported in 19 publications from Mainland China, Hongkong, and Taiwan (35, 55–72). Of them, 19 (54.3%) were female and 16 (45.7%) were male; the medium age at onset was 4 months. Thirty-two (91.4%) received BCG vaccination and 30 (93.8%) developed BCG disease. Among the 30 patients, 11 (36.7%) developed BCGitis, and 19 (63.3%) developed BCGosis.

Co-infections included EB virus, Staphylococcus epidermidis, Escherichia coli, Mycobacterium chelonae, Mycobacterium gordonae, Mycoplasma pneumoniae, Salmonella spp., and Herpes simplex virus (HSV) II. Twenty-one (60%) patients first presented with swollen lymph nodes in axilla, neck, or groin. Clinical manifestation varied depending on the gene mutation that impaired the IFN-γ-dependent immunity.

The following reports contain 9 genetic mutations and detailed clinical data of all the 65 Chinese MSMD patients, along with typical patients from other countries:

### 3.1 IFNGR1 Deficiency

Three Chinese Patients in Taiwan from two unrelated families were identified with a hotspot *IFNGR1* heterozygous deletion mutation (818del4) in exon 6 resulting in cutaneous granulomas and multiple osteomyelitides infected by NTM, MAC, and *Mycobacterium scrofulaceum* (62). The 818del4 mutation was also reported in a Chinese child who suffered from disseminated BCG disease and multi-system lesions (70). Three female patients who presented with enlarged lymph nodes under the left axilla were diagnosed with AD IFNGR1 deficiency. They carried heterozygous mutations in positions c.818-821deltTTAA, c.655G>A, and p.N274Hfs*2, respectively (58, 67, 71). Two patients with AR IFNGR1 deficiency and extensive BCG dissemination to the lung, intestinal tract, central nervous system, and bone were reported three months after birth. One case had a c.665G>A (p.G219R) homozygous mutation. Another case had c.665G>A (p.G219R) and c.310 C>A (p. A104N) compound heterozygous mutations (72). In addition to these nine patients reviewed, the Chinese cohort study reported another 5 IFNGR1 deficient patients, all of them presented with BCGosis, 3 with homozygous mutations, and 2 with compound heterozygous mutations (73).

Notably, the onset age of BCG-induced disease in all 14 Chinese patients with *IFNGR1* mutation was less than one year. In contrast, the mean onset age in American, Irish, and German cases was 13.4 years (15). Compared with AD deficient patients, those with AR deficiency were more likely to suffer from disseminated life-threatening infections, were less responsive to IFN-γ supplement therapy, and had higher mortality; these findings were similar to those of an earlier study (75).

### 3.2 IFNGR2 Deficiency

Two IFNGR2 deficient children were reported in China. A 3-month-old infant presented with an enlarged left axillary lymph node following BCG vaccination and was reported to have a heterozygous mutation of the *IFNGR2* gene in C.235c >A (58). Another child developed disseminated infections with complete IFNGR2 deficiency due to a frameshift mutation of IFNGR2 in exon 4 at position 514dupT (73).

Partial AR IFNGR2 deficiency is a milder disease than complete deficiency. It is more likely to present with milder infections and has a more favorable prognosis despite early onset (17, 76, 77). AR partial IFNGR2 deficiency has been reported in patients homozygous for c.958insT (77), p.S124F, p.G141R (78), p.R114C (79), p.G227R (80), c.1A>G, c.4delC mutations (81) around the world. AD partial IFNGR2 deficiency resulting from haplo-insufficiency has been reported in one Polish patient who had a mild form of localized BCG disease (17).

### 3.3 ISG15 Deficiency

In one report, three young children from a Chinese family presented with idiopathic basal ganglia calcification and intermittent seizure. They were diagnosed as AR ISG15-deficient, variant in exon 2 of ISG15: c.163C.T/163C.T (35).

### 3.4 IL12B (IL12p40) Deficiency

A 27-day-old Chinese female infant with IL12B deficiency presenting with BCGosis, pneumonia, and diarrhea was found lacking exon 3, but the splicing site of the mutation was not detected (61).

To our knowledge, there have been no more than 80 patients with IL12B deficiency reported all over the world (19,^82^–^85^). Most of them were characterized by childhood BCG or *Salmonella* infections, other infections included chronic mucocutaneous candidiasis, nocardiosis, and klebsiellosis. The average age at first infection in the 44 symptomatic patients was 1 year.

### 3.5 IL12RB1 Deficiency

Twenty-two patients with IL12RB1 mutations were identified in the Chinese cohort study (73). Seven were homozygous, the other 15 were compoundly heterozygous and 59.1% (13/22) carried a mutation at position 211. Lee PP et al (63) reported two patients, one suffered BCGosis with a novel nonsense mutation 853C>T (Q285X) in exon 9, and another one suffered BCGitis with a mutation 1791 + 2T>G. The heterozygous mutation of the second patient has also been discovered in two other Chinese patients (66, 67). Zhou XP et al (68) identified three novel compound heterozygous mutations in the *IL12RB1* gene (c.635G>A, c.765delG; c.632G>C, c.847C>T; c.64G>A, c.1673insGAGCTTCCTGAG) in three Chinese families with MSMD. One of the heterozygous mutations, c.632G > C, has been discovered in a Chinese patient from Taiwan who carried the homozygous mutation. He did not develop BCG-induced disease after receiving the BCG vaccine. He had recurrent *S. enteritidis* D sepsis and pneumatocele and carried a missense *IL12RB1* mutation (Arg211Pro) (62). Two male infants were diagnosed with BCGosis, Whole Exome Sequencing (WES) analysis showed IL-12RB1 heterozygous gene mutations; the mutations of c.339-340delCT, and p.L113Lfs*15 had not been reported previously (66). One 20-month-old female was diagnosed with BCGitis, she carried heterozygous missense mutations at site 632 of exon 7 (R211P) and site 1106 of exon 10 (I369T) (69). A 5-year-old boy who presented with repeated BCG infections in multiple organs carried heterozygous missense mutation (C.783 +1G>A) in exon 8 (61).

IL12RB1 mutation is the most common defect in MSMD. More than 250 patients with complete or partial AR or AD IL12RB1 deficiency have been reported (75, 86–88). The c.1791+2T>G mutation was the most frequent IL12RB1 mutation, found in more than 30 cases from 20 kindreds in Iran, China, Saudi Arabia, Spain, Turkey, Mexico, and France (63, 86, 88–92). Most cases occurred with early-onset, at a mean age of 2.4 years, with many in neonates receiving BCG immunization at birth. Besides BCGosis and NTM infection, half of the patients also developed invasive salmonellosis. Generally, IL-12RB1 deficiency is characterized by early-onset mycobacterial disease with rare recurrences and salmonellosis with frequent recurrence.

### 3.6 STAT1 Deficiency

Six cases of STAT1 deficiency were reported in China. Liu WQ (57) reported a BCGosis patient with AD STAT1 deficiency due to mutation c.1228A>G (P.K410E) in exon 15. Ying WJ et al (73) reported two patients who presented with recurrent ulceration and granulation hyperplasia at the BCG vaccination site. Two novel mutations in R378G and N460D were detected. Tan Y (58) described a 3-month-old BCGosis girl with abscesses in the left upper arm, a lung infection, and a spinal lesion. She was diagnosed with complete AD STAT1 deficiency with a heterozygous mutation of c.2071A>G in exon 23. Chen XM (59) and Fei L et al (60) reported 2 BCGosis cases: one had a mutation of R378T in DNA-binding, the other had two heterozygous mutations, c.1550A>G(p.D517G) and c.1127+4C>T.

Different forms of partial and complete STAT1 deficiency were identified in Germany, India, Japan, Saudi Arabia, Turkey, and elsewhere (23, 75, 93–95). Systematic reviews described 442 unique patients with STAT1 gain-of-function (GOF) mutations and 39 unique patients with STAT1 LOF mutations in various countries (96, 97). The STAT1 GOF mutations were often documented with chronic mucocutaneous candidiasis, lower respiratory tract infections, and autoimmune thyroid disease. The STAT1 loss-of-function (LOF) mutations most commonly manifested as MSMD, followed by osteomyelitis, and lymphadenopathy.

### 3.7 TYK2 Deficiency

The earliest report of a TYK2 gene mutation associated MSMD in China was published in 2016 (56). A 15-month-old boy suffered from repeated pulmonary infection, chronic otitis media, intractable eczema-like rash, repeated skin abscess, HSV infection, intracellular bacterial infection, and remarkedly increased total IgE. Analysis revealed compound heterozygous mutations of c.2269 C>G in No. 16 exon and c.149 delC in No. 3 exon in the *TYK2* gene. Yang Y et al (55) reported an adult MSMD patient with a completely AR *TYK2* gene mutation in 2020. This male developed pulmonary tuberculosis at 18-year-old and received anti-TB treatment. Two years later, he developed pulmonary lesions again, with erythema nodules on the extremities, and repeated fever. Molecular tests yielded positivity for *M. chelonae* and *M. gordonae* in the lower respiratory tract and revealed compound heterozygous mutations of c.997G>A in exon 7 and c.10C>T in exon 3 of the *TYK2* gene.

The first patient who was reported with inherited complete AR TYK2 deficiency was from Japan (29), he developed atopic dermatitis with a hyper-IgE syndrome and unusual susceptibility to various microorganisms, including non-typhoid *Salmonella* infections and recurrent cutaneous staphylococcal and mycobacterial infections. Subsequently, seven other TYK2-deficient patients who presented with mycobacterial and/or viral infections were reported from five families in Argentina, Iran, Morocco, and Turkey (30).

### 3.8 NEMO Deficiency

Only two NEMO deficiencies were reported from China. Li L (61) described a one-month-old male infant with a semisynthetic missense mutation c.1241T>A (p.V414D) in exon 10. His symptoms included cough, fever, and diarrhea, and he died five months later. Tan Y (58) reported a 3-month-old male infant with XR NEMO deficiency (heterozygous mutation in c.751G>C) who developed left axillary mass after BCG vaccination.

The first report of NEMO deficiency was in 1991 (98) and described two American brothers who presented with cutaneous *M. avium* complex infection; their nephew suffered from disseminated *M. avium* complex in 1994 (99) and another case from this family was noted in 2006 (27). Further investigation of this family confirmed the E315A mutation in NEMO. An R319Q mutation may also result in NEMO deficiency, as reported in two unrelated European boys from France and Germany (27). *IKK* gene-associated NEMO deficiency has presented with ectodermal dysplasia (100–102), and a complex progressive immunological syndrome characterized by an absence of sweat glands, sparse scalp hair, and rare conical teeth (103).

### 3.9 CYBB Deficiency

An 8-month-old Chinese infant was identified to have CYBB deficiency, after presenting with enlarged axillary lymph nodes and pneumonia (61). WES analysis found a mutation in mRNA315-917, but the splicing sites were not defined.

Bustamante (33) reported two kindreds in which otherwise healthy male adults developed XR MSMD syndromes. Four maternally related men of a French kindred were identified as suffering from mycobacterial diseases. One had a mutated *CYBB* allele encoding a Q231P substitution, and the other had a mutated *CYBB* allele encoding a T178P substitution.

## 4 Laboratory Tests

Cytokine production assay, flow cytometry-based tests targeting surface receptors/cytokines/phosphorylated STAT molecules, molecular detection, and other tests are widely used for the evaluation of MSMD in most countries including China.

### 4.1 Cytokine Production Assay

Enzyme-linked immunosorbent assay (ELISA) was used in the diagnosis of all reported Chinese MSMD patients. ELISA is a simple technique that can rapidly identify MSMD patients who have complete IFN-γ receptor deficiency by measuring baseline IFN-γ in plasma (104). The levels of IFN-γ in the plasma of healthy individuals and patients with various forms of MSMD are different. IFN-γ were almost undetectable in healthy individuals, partial dominant IFNGR1 deficiency and partial recessive IFNGR2 deficiency patients. Low levels of IFN-γ were detected in the plasma of partial recessive IFNGR1 deficiency patients. Very high levels of IFN-γ were seen in patients with complete IFNGR1 and IFNGR2 deficiency (104). Another study suggested that a very high baseline level of IFN-γ could reflect an acute mycobacterial disease, Thus, the infectious state of the patient needs to be considered, as baseline IFN-γ plasma levels may vary in acute infection compared with the convalescent phase (105, 106).

In addition, ELISA assay for measurement of IL-12p40, IL-12p70 and IFN-γ after stimulation of whole blood or peripheral blood mononuclear cells (PBMCs) with stimulation conditions comprising live BCG stimulation at a multiplicity of infection of 20 (BCG/leukocytes) with or without hrIL-12p70 (20ng/mL), or hrIFN-γ (5000 IU/mL) co-stimulation for 18h (for IL-12 measurement) or 48h (for IFN-γ and IL-12 measurements), could be used to differentiate between immune defects due to IFN-γ production or IFN-γ response (9).

Besides ELISA assay, Luminex and flow cytometry (which will be discussed below) could be used to determine MSMD-related cytokines’ production.

### 4.2 Flow Cytometric Evaluation of Immunological Function

Flow cytometric evaluation is also widely used in the early diagnosis of MSMD, especially in patients deficient in surface expression of biomarkers such as IFNGR1, IFNGR2, IL-12RB1 and IL-12RB2. It is helpful in the rapid diagnosis of an underlying genetic defect by assessing cytokine expression levels of IL-12p40, IL-12p70, and IFN-γ from patients’ PBMCs. The flow cytometry-based assay of cytokine production can also distinguish between IFN-γ response defects and IFN-γ production defects (106). With complete IFN-γ response defects, there is no response to recombinant human IFN-γ in terms of IL-12 production, whereas, in partial IFN-γ response defects, the response is impaired in a dose-dependent manner but not abolished (107). In addition, the phosphorylation of intracellular STAT proteins (STAT1/STAT3/STAT4) could also be determined by flow cytometry assay, which helps define which pathways contributed to the immune defects in MSMD patients. There were also unique microbial infections in MSMD patients from China not seen in other cohorts. For instance, infection with Talaromyces marneffei has been reported in patients with functional defect in interleukin-12/interferon-γ axis. Application of flow cytometry in the diagnostics pipeline for these patients with disseminated talaromycosis is simple and rapid. STAT1 hyperphosphorylation in response to IFN-α or IFN-γ and delayed dephosphorylation is diagnostic for gain-of-function STAT1 disorder, while absent STAT1 phosphorylation in response to IFN-γ but normal response to IFN-α is suggestive of IFN-γ receptor deficiency (108).

### 4.3 Molecular Detection

Confirmation of these MSMD cases mainly relies on molecular detections, including Sanger sequencing, next-generation sequencing (NGS), WES, or whole-genome sequencing (WGS).

Sanger sequencing is a good option for further clarifying whether MSMD exists, and is an excellent diagnostic or research process if the patient’s phenotype is typical of known specific genotypes. NGS has made it possible to decipher unknown disease-causing mutations in multiple unrelated individuals, leading to new insights into disease mechanisms (109) and can be time-saving (110). It is suitable when the patient’s phenotype is consistent with the known disease-causing genetic defects. However, a large number of patients with suspected MSMD carry unknown disease-causing genes (111). For a full diagnosis of a PID and genetic counseling, WES or WGS may be more useful. WES is currently the most reasonable approach. If a mono- or bi-allelic genotype is known to cause the patient’s phenotype based on previous studies, a diagnosis can be made easily. WGS has entered diagnostic strategies for newborns with suspected PID (112). It has clear potential technical benefits over WES as it can detect intronic and intergenic mutations and provides uniform coverage (110).

Besides the laboratory tests mentioned above, there are other assay might facilitate MSMD diagnosis, such as detection of IFN-γ autoantibodies by using an ELISA system, observation of IFN-γ level recovery after the addition of exogenous IFN-γ to patient serum (113–115), detection of respiratory burst defects for MSMD patients with CYBB deficiencies (33), and assessment of CD11c+CD1c+ blood myeloid dendritic cells for patients with partial AD IRF8 deficiency (31).

In conclusion, the widespread availability of NGS/WES/WGS detections in most countries including China offers an opportunity to diagnosing MSMD with exact genetic variants in the previously reported genes, the functional tests such as cytokine production assay and flow cytometry-based assay are important to seek and define novel variants in a large number of unclassified or undetermined significance detected by these high-throughput sequencing technologies. As mentioned above, 60% of patients with clinical manifestations suggestive of MSMD lack a defined genetic cause, the findings of novel genetic etiologies of MSMD are important for diagnosing suspected MSMD patients in the fastest and most convenient way, particularly in countries where BCG vaccination is widespread, such as China. It enables the establishment of specific therapeutic measures that will improve the patient’s prognosis and quality of life.

## 5 Treatment Approach

### 5.1 Anti-Infection

Among all of the 65 Chinese MSMD patients characterized to date, 92.3% (60/65) developed BCG disease after the routine childhood BCG vaccine inoculation, so anti-mycobacterial treatment (ATT) is the most recommended treatment approach. All 30 MSMD patients from the Chinese cohort study (73) received ATT, and the 14 MSMD patients in the 19 literature reports received ATT therapy (**Table 3**). Patients with BCGitis started on ATT with a combination of three drugs: isoniazid (INH), rifampicin (RFP), and ethambutol (EMB). Pyrazinamide (PZA) was not part of the standard treatment as BCG has inherent PZA resistance (116). However, low-level INH resistance was observed in the Denmark strain (SSI 1331) and Connaught strain (117, 118), so BCG culture and sensitivity results could help to optimize the ATT regimen. Patients with BCGosis should be given four or more anti-TB drugs until complete recovery. The second line anti-TB drugs such as Linezolid, a quinolone (e.g. ciprofloxacin, levofloxacin), an aminoglycoside (e.g. amikacin, streptomycin), and clarithromycin should always be considered regarding the degree of dissemination. After infections have been controlled, a prophylactic regimen with two drugs (usually INH plus RFP) should be continued (119). ATT therapy may have to be continued for longer periods than for average TB diseases, possibly even for life (120). Besides ATT therapy, other anti-infective and anti-viral drugs can be selected according to the type of infection.

**Table 3:** Treatment and prognosis of the 65 MSMD patients reported in China.

	Group A (n=30)	Group B (n=35)	Total (n=65)
**Treatment**			
ATT therapy	30	14	44 (67.7%)
IFN-γ therapy	21	9	30 (46.2%)
HSCT therapy	2	0	2 ( 3%)
**Outcome**			
**Died**	**5**	**7**	**12 (18.5%) n**
*IL-12RB1*	3	3	6 (9.2%)
*NEMO*	–	1	1 (1.5%)
*IL12B*	–	1	1 (1.5%)
*ISG15*	–	1	1 (1.5%)
*IFNGR1*	2	1	3 (4.6%)
**Survived**	**22**	**21**	**43 (66.2%) n**
*TYK2*	–	2	2 (3.1%)
*STAT1*	2	2	4 (6.2%)
*IL-12RB1*	16	8	24 (36.9%)
*IFNGR1*	3	6	9 (13.4%)
*IFNGR2*	1	–	1 (1.5%)
*CYBB*	–	1	1 (1.5%)
*ISG15*	–	2	2 (3.1%)
**Lost/UNK**	**3**	**7**	**10 (15.4%) n**

Group A: 30 MSMD patients from the Chinese cohort study; Group B: 35 MSMD patients from the 19 publications we have reviewed; UNK: unknown (not mentioned in the literature).

### 5.2 IFN-γ Therapy

As shown in **Table 3**, 46.2% (30/65) of Chinese MSMD patients had received IFN-γ therapy, and most of them showed improvement. IFN-γ therapy was only effective for patients whose cellular responses to IFN-γ were intact, or impaired but not abolished. Patients suffering from AR complete IFNGR1 or IFNGR2 deficiency do not respond to IFN-γ therapy (111). Accordingly, IFN-γ should be given to patients only after evaluating their IFN-γ responses. The initial regimen should use a dose of 30-50 mg/m^2^ administered subcutaneously thrice a week. If the patient fails to respond to this regimen, the IFN-γ dose should be increased step-wise at monthly intervals until a response is finally observed or up to a maximum dose of 400 mg/m^2^, thrice a week (99).

### 5.3 HSCT

If IFN-γ would be ineffective, such as in the absence of specific functional receptors in AR complete IFNGR1 and IFNGR2 deficiency, allogeneic HSCT will be the only curative treatment. There were only 2 of the 65 Chinese MSMD patients who presented with IFNGR1 deficiency and had received HSCT therapy after failed treatment with IFN-γ. One died from rejection and severe infection after the transplant, and another survived for 5 months (73).

So far, the overall prognosis of HSCT for patients suffering from IFNGR1 or IFNGR2 deficiency is poor (121). The main reasons for the low success rate are recurrent infections during the transplantation scenario, the risks of graft rejection, and graft versus host disease (GVHD). An unusually high graft rejection rate is associated with the high IFN-γ serum levels that are presented in IFNGR–deficient MSMD patients as a result of failure to clear IFN-γ from the blood (122).

### 5.4 Gene Therapy

Hematopoietic stem cell gene therapy (HSCGT) has progressed over decades from ineffectiveness to become a promising therapeutic success for various PID. Approaches to autologous transplant/gene therapy using lentiviral vectors have produced clinical benefits similar to those attained after allogeneic transplants treating several disorders (123). A recent study presented the first gene therapy approach for patients suffering from AR complete IFN-γR1 deficiency. Lentiviral vectors were used to correct defective IFN-γ-mediated immunity without generating GVHD (124), and we expect HSCGT to become an increasingly effective method to cure PID patients in the future.

Developments in gene-editing procedures, which include gene disruption, gene correction, and gene insertion, are leading toward application for *ex vivo* gene correction in HSCGT. Such application may have advantages compared to integrating genes added by viral vectors (125, 126). A good deal of research is directed at modifying patient stem cells, but it is time-consuming, expensive, and still at an early stage of development (125). To our knowledge, HSCGT has not been introduced in clinical practice for treating MSMD in China.

## 6 Prognosis

As shown in **Table 3**, 18.5% (12 of 65) Chinese MSMD patients have died of severe infections, 66.2% (43 of 65) survived, and 15.4% (10 of 65) were lost to follow-up or their progress remains unreported in the literature. Six of 31 IL12RB1 deficient patients died. Among the 24 surviving patients with IL12RB1 deficiency, the mortality was significantly lower in those who were treated with IFN-γ than in those not treated with IFN-γ. Three patients with IFNGR1 deficiency died, of whom 2 had received HSCT, one died of a severe infection 6 months after HSCT, and the other received HSCT and was well after 5 months. Three other patients, who were IL12B, ISG15, and IFNGR1 deficient, died of severe disseminated infections or ATT. Overall, among the 30 patients, 27 were followed up or had died by the time of publication. Follow-up information in the cohort study is more complete as it was in a prospective study (73), and the median survival time was 26 months (range from 5 to 173 months).

The prognosis depends on the specific underlying mutation of the genes involved in the IFN-γ-mediated immunity. Consistent with international reports (127, 128), IL12RB1 deficiency and complete IFNGR1 deficiency were the two types in China with the highest morbidity and mortality. Patients who were actively treated with anti-infection therapy, including IFN-γ therapy, had a better prognosis than those who were not actively treated.

## 7 Discussion

MSMD can be regarded as an infection syndrome that is mainly determined by the patient’s genetic make-up. Over the past two decades, it has been shown to encompass defects in 19 genes primarily involved in the production of, or response to, IFN-γ/IL-12/IL-23. These genetic dissections of patients with MSMD have shed light on the cellular and molecular basis of human immunity against mycobacteria. However, the genetic puzzle of failed immunity to mycobacterial infections remains far from solved, since no genetic etiology has yet been identified for almost 60% of patients with MSMD.

IL-12RB1 deficiency was the predominant gene affected in China, the c.1791+2T>G mutation was the most frequent IL12RB1 mutation, which was consistent with the reports of other countries. The novel mutations of Q285X, c.339-340delCT, p.L113Lfs*15, c.635G>A, c.765delG, c.632G>C, c.847C>T, c.64G>A, c.1673insGAGCTTCCTGAG in IL-12RB1 gene had not been reported previously. Three ISG15 deficiency patients who had mutation of c.163C.T/163C.T were first reported. Other novel mutations, such as c.1228A>G of STAT1 gene, p.A104N of IFNGR1 gene, p.V414D of NEMO gene, were all first reported.

The incidence of MSMD was found to be closely related to consanguineous marriages. Along with a large number of consanguineous marriages in the Middle-Eastern countries, such as Saudi Arabia, Iraq, Syria, Iran, Turkey, and Morocco, MSMD was reported to be more frequent in childhood mycobacterial disease than in other developed countries (129). MSMD was mainly thought to be AR because of the high frequency of both multiple-case sibships and consanguineous kindreds (130, 131). We found no significant difference in incidence or manifestations between China and the non-consanguineous marriages regions.

BCG-related complications were the major presenting manifestations in MSMD in 4–80% of the patients (132). In China, as the BCG vaccination covered over 99% of infants, BCG infection often presented as the index symptom of MSMD at an early age. Therefore, the clinical characteristics, diagnosis, treatment, and prognosis could be different from cases in regions where BCG vaccination is not provided. This study has provided an up-to-date literature review involving all eligible Chinese publications. Through this study, the onset age of MSMD in most Chinese patients was less than one year. BCGosis is the most typical manifestation in MSMD cases reported in China, followed by BCGitis; 41 of 65 patients (63.1%) developed BCGosis, and 19 of 65 patients (29.2%) developed BCGitis. These findings may contribute to domestic consensus on managing MSMD or BCG disease and raise awareness of screening MSMD in infants with abnormal responses to BCG vaccination.

Except for BCG-related complications, multiplex kindreds with MSMD were susceptible to affect with environmental NTM, *Mtb*, Salmonella and many other pathogens. Salmonella occurred in about half the reported patients. Among all of the 65 Chinese MSMD patients, co-infections included Salmonella, NTM (Mycobacterium chelonae, Mycobacterium gordonae, Mycobacterium scrofulaceum, MAC), EB virus, Staphylococcus epidermidis, Escherichia coli, Mycoplasma pneumoniae, klebsiella, HSVII, Candida albicans, and so on. The severity of the clinical manifestations depended on the underlying mutation of genes involved in the IFN-γ-mediated immunity. The various clinical manifestations ranged from recurrent localized disease to progressive disseminated infections (15, 75).

Overall, the available data has shown that IFN-γ-mediated immunity is crucial to anti-mycobacterial defense. Novel genetic etiologies in patients with MSMD are likely to reflect a connection to IFN-γ-mediated anti-mycobacterial immunity, however, it remains possible that not all MSMD patients will be linked to IFN-γ signaling. Currently, the development of NGS, including both WGS and WES, could be used to improve the screening for novel mutations that are not easily detectable by the traditional Sanger sequencing. Such work will lead to the discovery of more genetic etiologies, and accelerate genetic dissection in MSMD patients who are currently without a genetic diagnosis. In addition, the discovery of novel genetic etiologies of MSMD will yield a better understanding of the pathogenesis and have important diagnostic and therapeutic implications. This will be especially important in regions endemic to TB and infections with other intracellular microbes. An improved possibility of offering genetic counseling to family members and specific treatments targeted at the patient’s immune system

## Author Contributions

LX, X-HL, and Z-DH participated in the study design, collected data, performed data analyses, drafted the manuscript, and takes responsibility for the manuscript as a whole. YY provided editing and data analysis. DL, X-YF, TL, and S-HL reviewed and revised the manuscript. All authors contributed to the article and approved the submitted version.

## Funding

This work was supported by the Shenzhen Key Medical Discipline Construction Fund (SZGSP010), the National Key Research and Development Program of China (2021YFC2301503), the National Natural and Science Foundation of China (81873884, 82171739, 81770011, 81900005), and the Shanghai Municipal Medical and Health Excellent Young Talents Training Program (GWV-10.2-YQ01).

## Conflict of Interest

The authors declare that the research was conducted in the absence of any commercial or financial relationships that could be construed as a potential conflict of interest.

## Publisher’s Note

All claims expressed in this article are solely those of the authors and do not necessarily represent those of their affiliated organizations, or those of the publisher, the editors and the reviewers. Any product that may be evaluated in this article, or claim that may be made by its manufacturer, is not guaranteed or endorsed by the publisher.

## Abbreviations

MSMD, Mendelian susceptibility to mycobacterial disease; PID, Primary immunodeficiencies; NTM, Non-tuberculous mycobacteria; TB, Tuberculosis; *Mtb*, *Mycobacterium tuberculosis*; AD, Autosomal dominant; AR, Autosomal recessive; XR, X-linked recessive; HSV*, Herpes Simplex Virus*; BCG, Bacillus Calmette-Guerin; BCGitis, Regional lymphadenitis; BCGosis, Disseminated BCG infection; CGD, Chronic granulomatous disease; GOF, Gain-of-function; LOF, Loss-of-function; IFN-γ, Interferon gamma; IL, Interleukin; WES, Whole exome sequencing; NGS, Next-generation sequencing; WGS, Whole genome sequencing; MAC, *Mycobacterium avium complex*; NTF, N-terminal fragment; HLA, Human leukocyte antigen; ELISA, Enzyme-linked immunosorbent assay; PBMCs, Peripheral blood mononuclear cells; ATT, Anti-mycobacterial treatment; INH, Isoniazid; RFP, Rifampicin; EMB, Ethambutol; PZA, Pyrazinamide; HSCT, Hematopoietic stem cell transplantation; HSCGT, Hematopoietic stem cell gene therapy; GVHD, Graft versus host disease.
